# Supplementary material for: NSMCE2, a novel super-enhancer-regulated gene, is linked to poor prognosis and therapy resistance in breast cancer
Source: BMC Cancer. 2022 Oct 12;22:1056. doi: 10.1186/s12885-022-10157-7 (PMC9555101; doi:10.1186/s12885-022-10157-7)
Supplement: Supplementary file 2 — Additional file 2. [file 12885_2022_10157_MOESM2_ESM.pptx]

## Slide 1
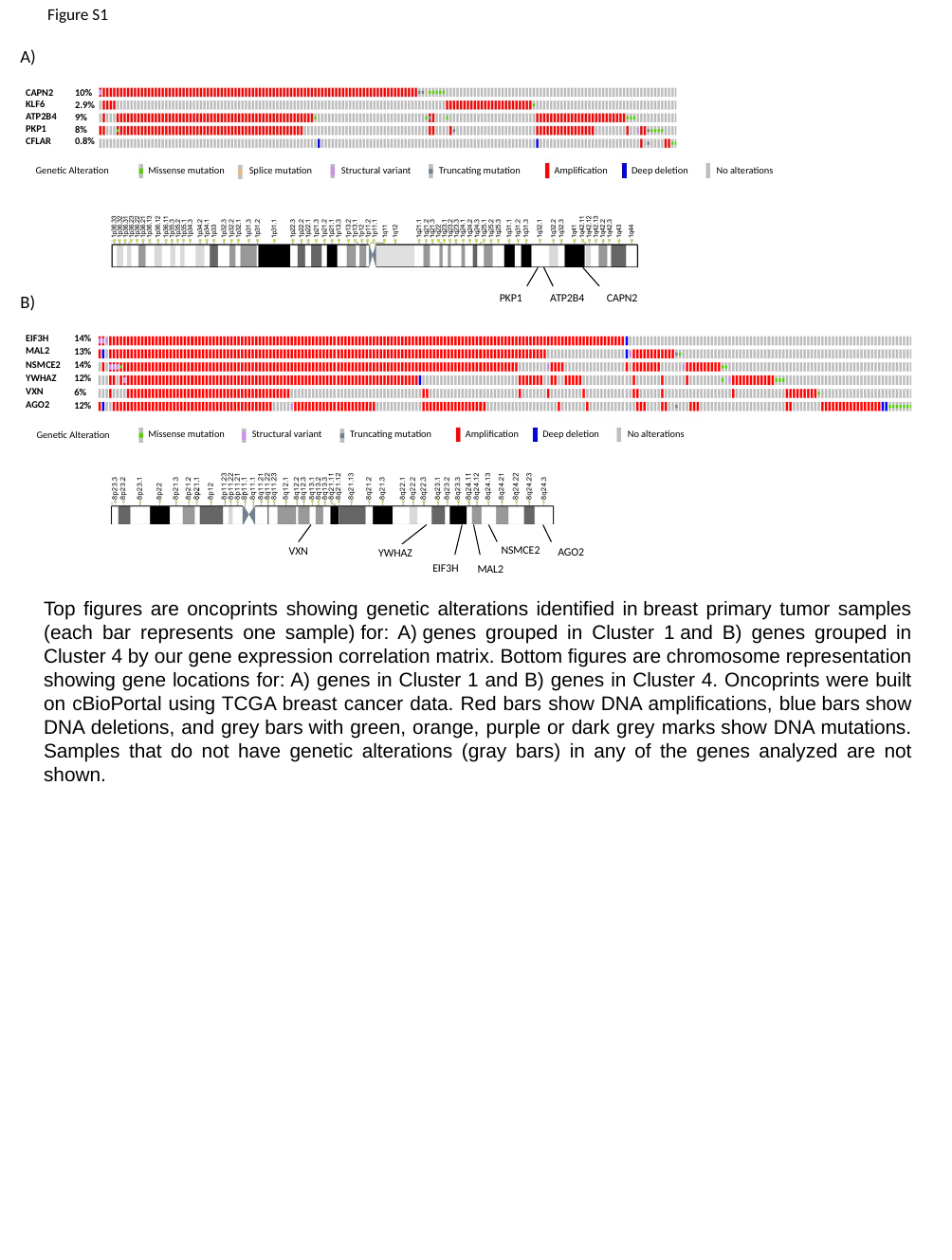

Figure S1
A)
CAPN2
KLF6
ATP2B4
PKP1
CFLAR
10%
2.9%
9%
8%
0.8%
Missense mutation
Splice mutation
Structural variant
Truncating mutation
Amplification
Deep deletion
No alterations
Genetic Alteration
PKP1
CAPN2
ATP2B4
B)
EIF3H
MAL2
NSMCE2
YWHAZ
VXN
AGO2
14%
13%
14%
12%
6%
12%
Missense mutation
Structural variant
Truncating mutation
Amplification
Deep deletion
No alterations
Genetic Alteration
NSMCE2
VXN
AGO2
YWHAZ
EIF3H
MAL2
Top figures are oncoprints showing genetic alterations identified in breast primary tumor samples (each bar represents one sample) for: A) genes grouped in Cluster 1 and B) genes grouped in Cluster 4 by our gene expression correlation matrix. Bottom figures are chromosome representation showing gene locations for: A) genes in Cluster 1 and B) genes in Cluster 4. Oncoprints were built on cBioPortal using TCGA breast cancer data. Red bars show DNA amplifications, blue bars show DNA deletions, and grey bars with green, orange, purple or dark grey marks show DNA mutations. Samples that do not have genetic alterations (gray bars) in any of the genes analyzed are not shown.
