# Supplementary material for: NSMCE2, a novel super-enhancer-regulated gene, is linked to poor prognosis and therapy resistance in breast cancer
Source: BMC Cancer. 2022 Oct 12;22:1056. doi: 10.1186/s12885-022-10157-7 (PMC9555101; doi:10.1186/s12885-022-10157-7)
Supplement: Supplementary file 3 — Additional file 3. [file 12885_2022_10157_MOESM3_ESM.pptx]

## Slide 1
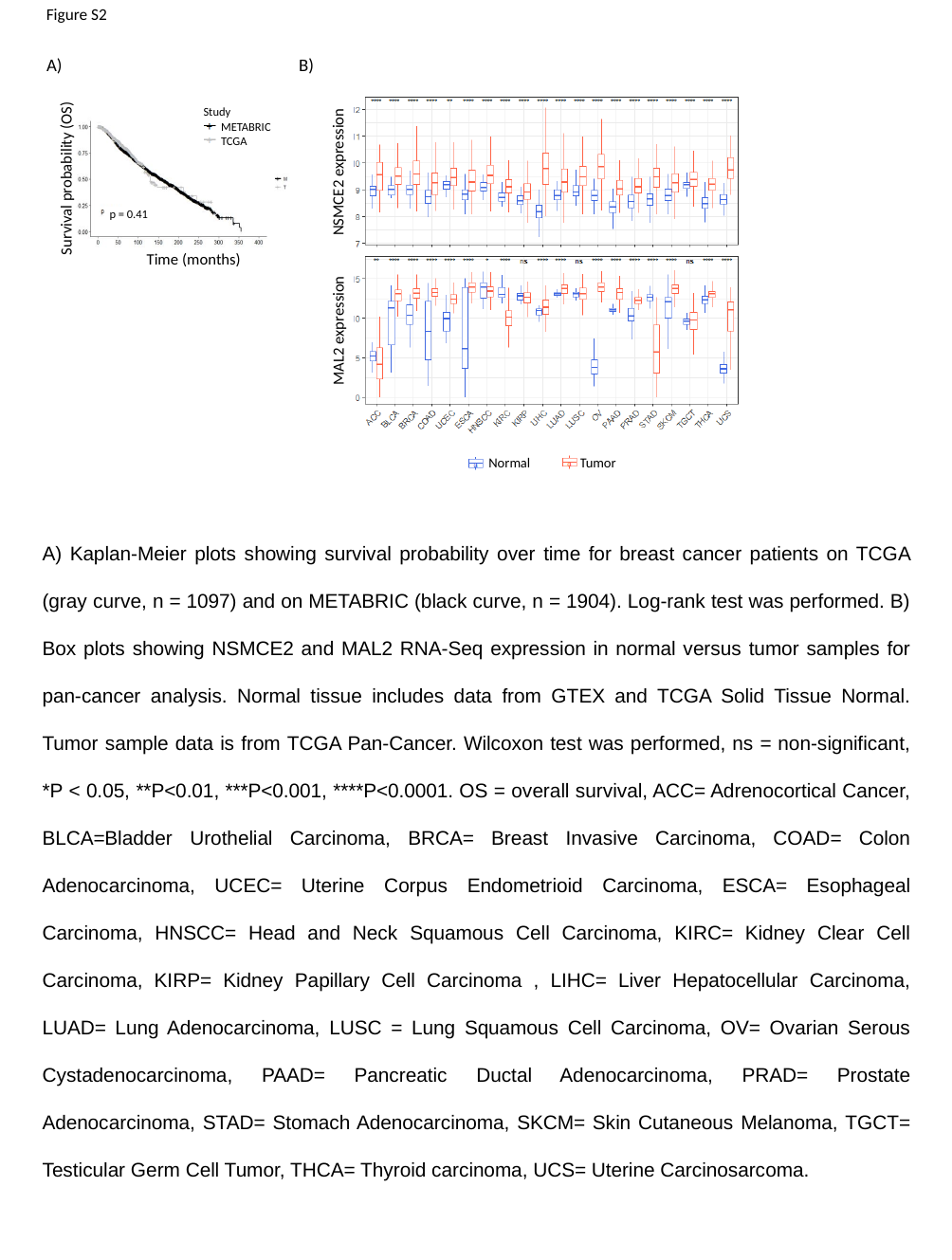

Figure S2
A)
B)
Study
METABRIC
TCGA
Survival probability (OS)
Time (months)
p = 0.41
NSMCE2 expression
MAL2 expression
Tumor
Normal
A) Kaplan-Meier plots showing survival probability over time for breast cancer patients on TCGA (gray curve, n = 1097) and on METABRIC (black curve, n = 1904). Log-rank test was performed. B) Box plots showing NSMCE2 and MAL2 RNA-Seq expression in normal versus tumor samples for pan-cancer analysis. Normal tissue includes data from GTEX and TCGA Solid Tissue Normal. Tumor sample data is from TCGA Pan-Cancer. Wilcoxon test was performed, ns = non-significant, *P < 0.05, **P<0.01, ***P<0.001, ****P<0.0001. OS = overall survival, ACC= Adrenocortical Cancer, BLCA=Bladder Urothelial Carcinoma, BRCA= Breast Invasive Carcinoma, COAD= Colon Adenocarcinoma, UCEC= Uterine Corpus Endometrioid Carcinoma, ESCA= Esophageal Carcinoma, HNSCC= Head and Neck Squamous Cell Carcinoma, KIRC= Kidney Clear Cell Carcinoma, KIRP= Kidney Papillary Cell Carcinoma , LIHC= Liver Hepatocellular Carcinoma, LUAD= Lung Adenocarcinoma, LUSC = Lung Squamous Cell Carcinoma, OV= Ovarian Serous Cystadenocarcinoma, PAAD= Pancreatic Ductal Adenocarcinoma, PRAD= Prostate Adenocarcinoma, STAD= Stomach Adenocarcinoma, SKCM= Skin Cutaneous Melanoma, TGCT= Testicular Germ Cell Tumor, THCA= Thyroid carcinoma, UCS= Uterine Carcinosarcoma.
