# Supplementary material for: NSMCE2, a novel super-enhancer-regulated gene, is linked to poor prognosis and therapy resistance in breast cancer
Source: BMC Cancer. 2022 Oct 12;22:1056. doi: 10.1186/s12885-022-10157-7 (PMC9555101; doi:10.1186/s12885-022-10157-7)
Supplement: Supplementary file 4 — Additional file 4. [file 12885_2022_10157_MOESM4_ESM.pptx]

## Slide 1
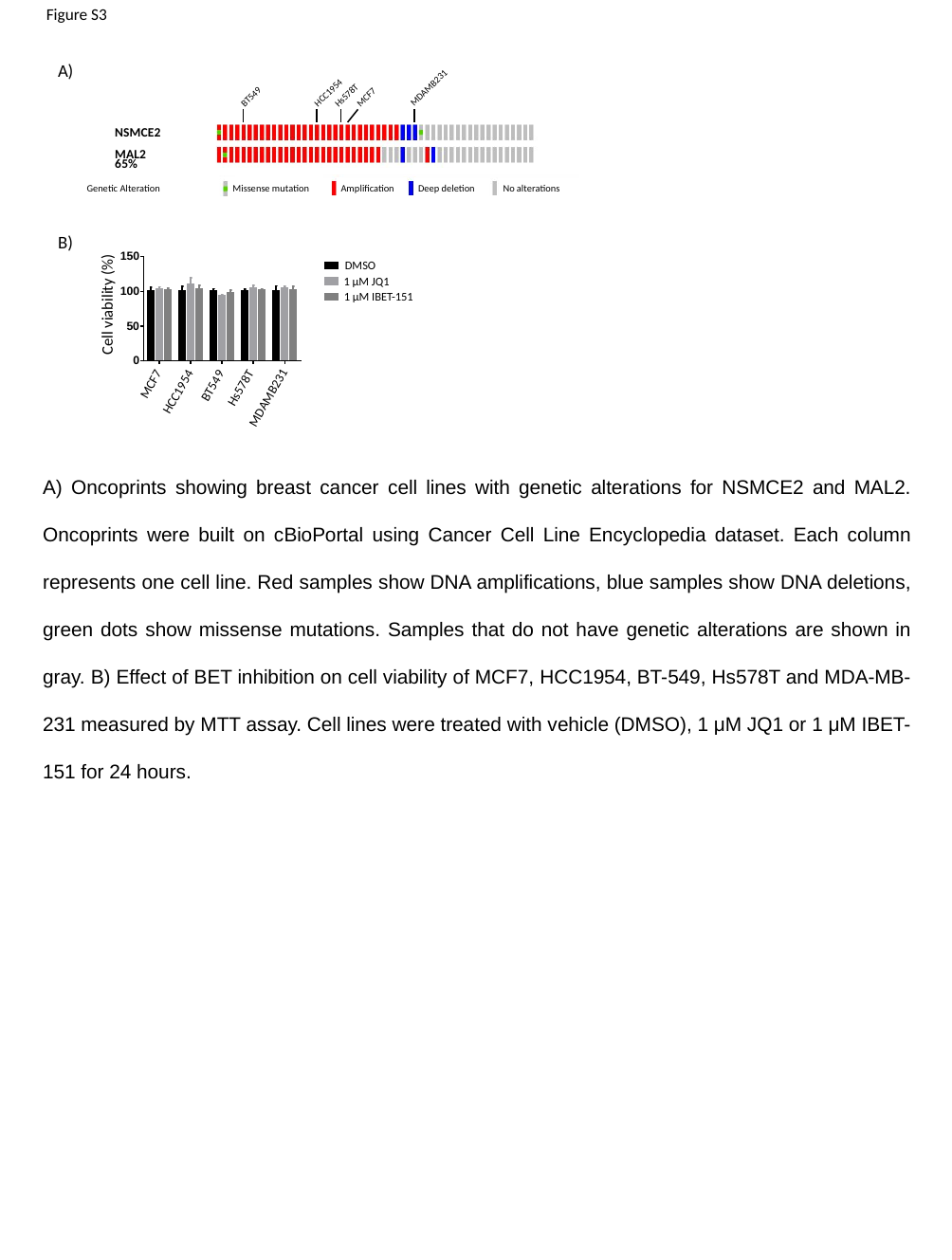

Figure S3
A)
MDAMB231
MCF7
Hs578T
HCC1954
BT549
NSMCE2	65%
MAL2	58%
Genetic Alteration
Missense mutation
Amplification
Deep deletion
No alterations
B)
Cell viability (%)
MCF7
HCC1954
BT549
Hs578T
MDAMB231
DMSO
1 µM JQ1
1 µM IBET-151
A) Oncoprints showing breast cancer cell lines with genetic alterations for NSMCE2 and MAL2. Oncoprints were built on cBioPortal using Cancer Cell Line Encyclopedia dataset. Each column represents one cell line. Red samples show DNA amplifications, blue samples show DNA deletions, green dots show missense mutations. Samples that do not have genetic alterations are shown in gray. B) Effect of BET inhibition on cell viability of MCF7, HCC1954, BT-549, Hs578T and MDA-MB-231 measured by MTT assay. Cell lines were treated with vehicle (DMSO), 1 μM JQ1 or 1 μM IBET-151 for 24 hours.
