# Supplementary material for: NSMCE2, a novel super-enhancer-regulated gene, is linked to poor prognosis and therapy resistance in breast cancer
Source: BMC Cancer. 2022 Oct 12;22:1056. doi: 10.1186/s12885-022-10157-7 (PMC9555101; doi:10.1186/s12885-022-10157-7)
Supplement: Supplementary file 5 — Additional file 5. [file 12885_2022_10157_MOESM5_ESM.pptx]

## Slide 1
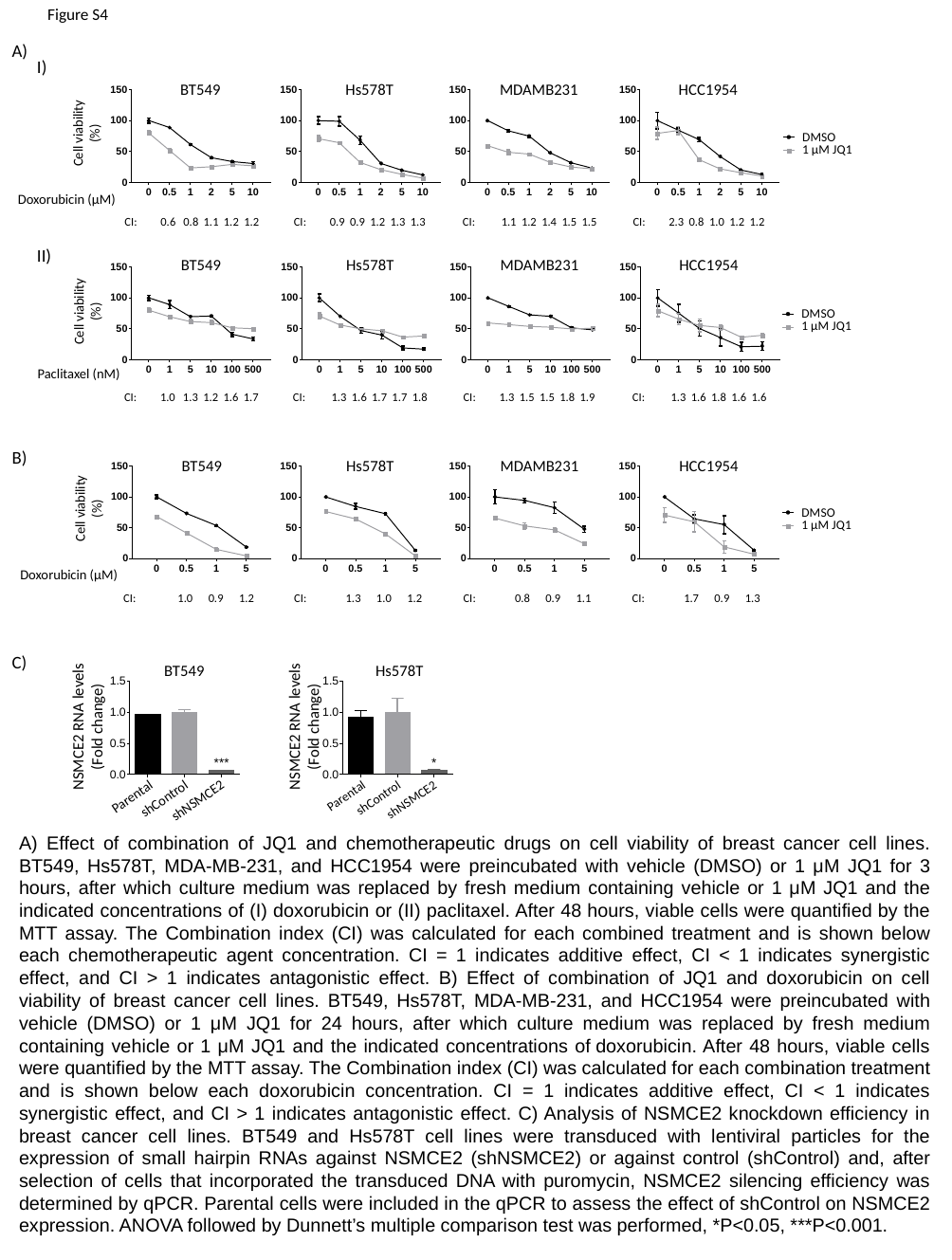

Figure S4
A)
I)
BT549
Hs578T
MDAMB231
HCC1954
Cell viability (%)
DMSO
1 µM JQ1
Doxorubicin (µM)
CI: 0.6 0.8 1.1 1.2 1.2
CI: 0.9 0.9 1.2 1.3 1.3
CI: 1.1 1.2 1.4 1.5 1.5
CI: 2.3 0.8 1.0 1.2 1.2
II)
BT549
Hs578T
MDAMB231
HCC1954
Cell viability (%)
DMSO
1 µM JQ1
Paclitaxel (nM)
CI: 1.0 1.3 1.2 1.6 1.7
CI: 1.3 1.6 1.7 1.7 1.8
CI: 1.3 1.5 1.5 1.8 1.9
CI: 1.3 1.6 1.8 1.6 1.6
B)
BT549
Hs578T
MDAMB231
HCC1954
Cell viability (%)
DMSO
1 µM JQ1
Doxorubicin (µM)
CI: 1.0 0.9 1.2
CI: 1.3 1.0 1.2
CI: 0.8 0.9 1.1
CI: 1.7 0.9 1.3
C)
NSMCE2 RNA levels (Fold change)
Parental
shControl
shNSMCE2
BT549
NSMCE2 RNA levels (Fold change)
Parental
shControl
shNSMCE2
Hs578T
A) Effect of combination of JQ1 and chemotherapeutic drugs on cell viability of breast cancer cell lines. BT549, Hs578T, MDA-MB-231, and HCC1954 were preincubated with vehicle (DMSO) or 1 μM JQ1 for 3 hours, after which culture medium was replaced by fresh medium containing vehicle or 1 μM JQ1 and the indicated concentrations of (I) doxorubicin or (II) paclitaxel. After 48 hours, viable cells were quantified by the MTT assay. The Combination index (CI) was calculated for each combined treatment and is shown below each chemotherapeutic agent concentration. CI = 1 indicates additive effect, CI < 1 indicates synergistic effect, and CI > 1 indicates antagonistic effect. B) Effect of combination of JQ1 and doxorubicin on cell viability of breast cancer cell lines. BT549, Hs578T, MDA-MB-231, and HCC1954 were preincubated with vehicle (DMSO) or 1 μM JQ1 for 24 hours, after which culture medium was replaced by fresh medium containing vehicle or 1 μM JQ1 and the indicated concentrations of doxorubicin. After 48 hours, viable cells were quantified by the MTT assay. The Combination index (CI) was calculated for each combination treatment and is shown below each doxorubicin concentration. CI = 1 indicates additive effect, CI < 1 indicates synergistic effect, and CI > 1 indicates antagonistic effect. C) Analysis of NSMCE2 knockdown efficiency in breast cancer cell lines. BT549 and Hs578T cell lines were transduced with lentiviral particles for the expression of small hairpin RNAs against NSMCE2 (shNSMCE2) or against control (shControl) and, after selection of cells that incorporated the transduced DNA with puromycin, NSMCE2 silencing efficiency was determined by qPCR. Parental cells were included in the qPCR to assess the effect of shControl on NSMCE2 expression. ANOVA followed by Dunnett’s multiple comparison test was performed, *P<0.05, ***P<0.001.
